# Supplementary material for: In Silico Drug Design of Anti-Breast Cancer Agents
Source: Molecules. 2023 May 18;28(10):4175. doi: 10.3390/molecules28104175 (PMC10223712; doi:10.3390/molecules28104175)
Supplement: Supplementary file 1 [file molecules-28-04175-s001.zip › molecules-2312448-supplementary.pdf]

2D & 3D interactions for top 10 compounds

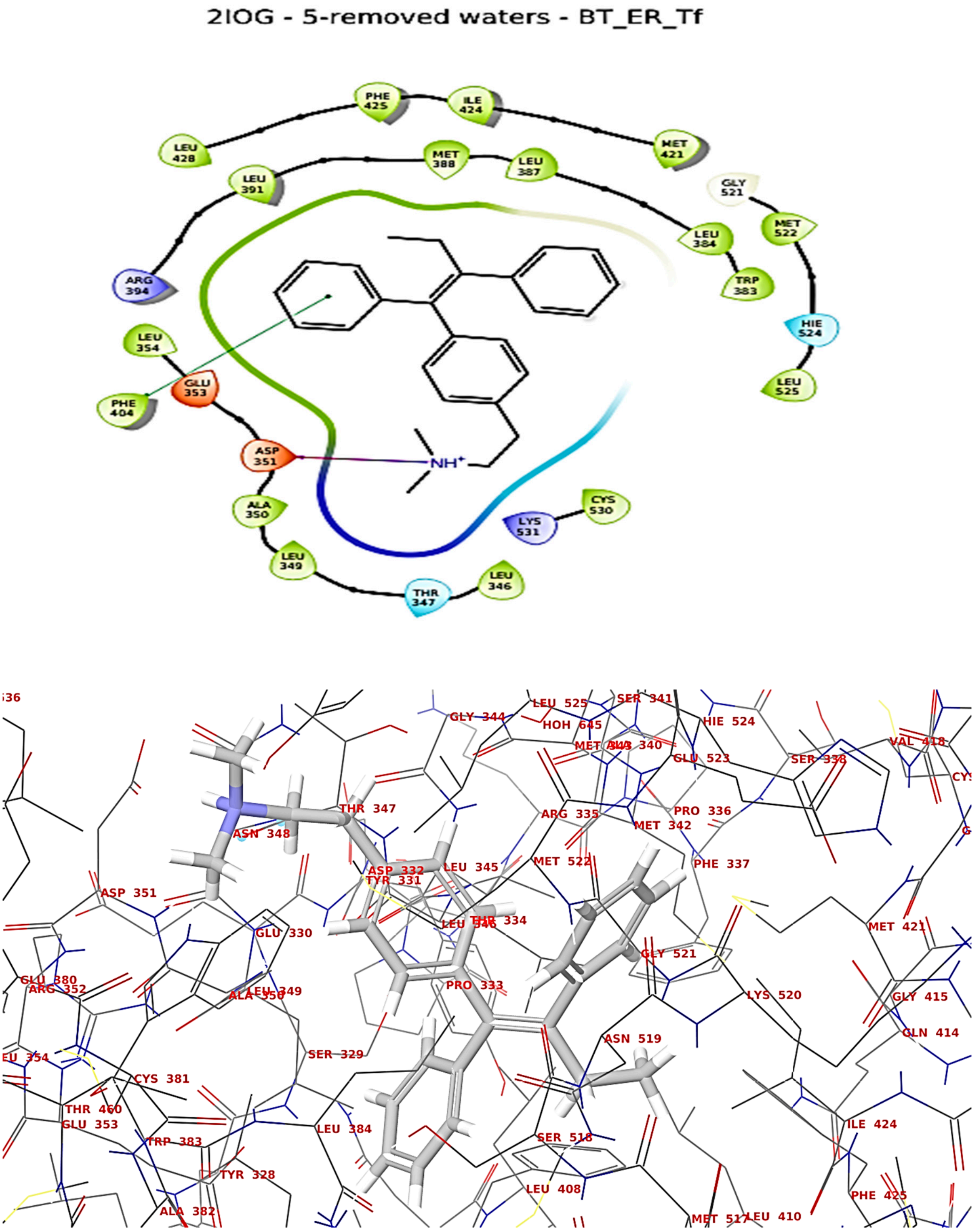

Figure S1a: 2D & 3D interactions for Compound BT ER Tf (Docking score -13.560)

2IOG - 5-removed waters - BT_ER_21b

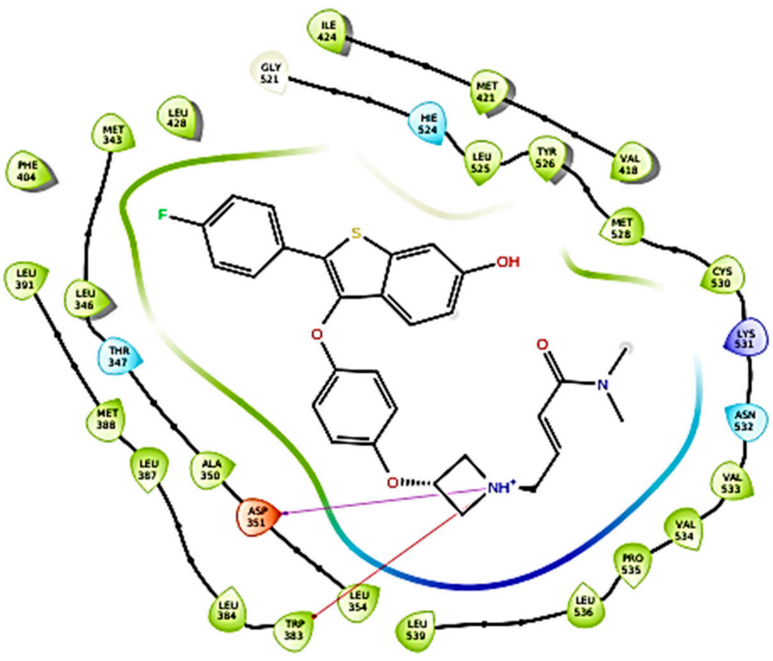

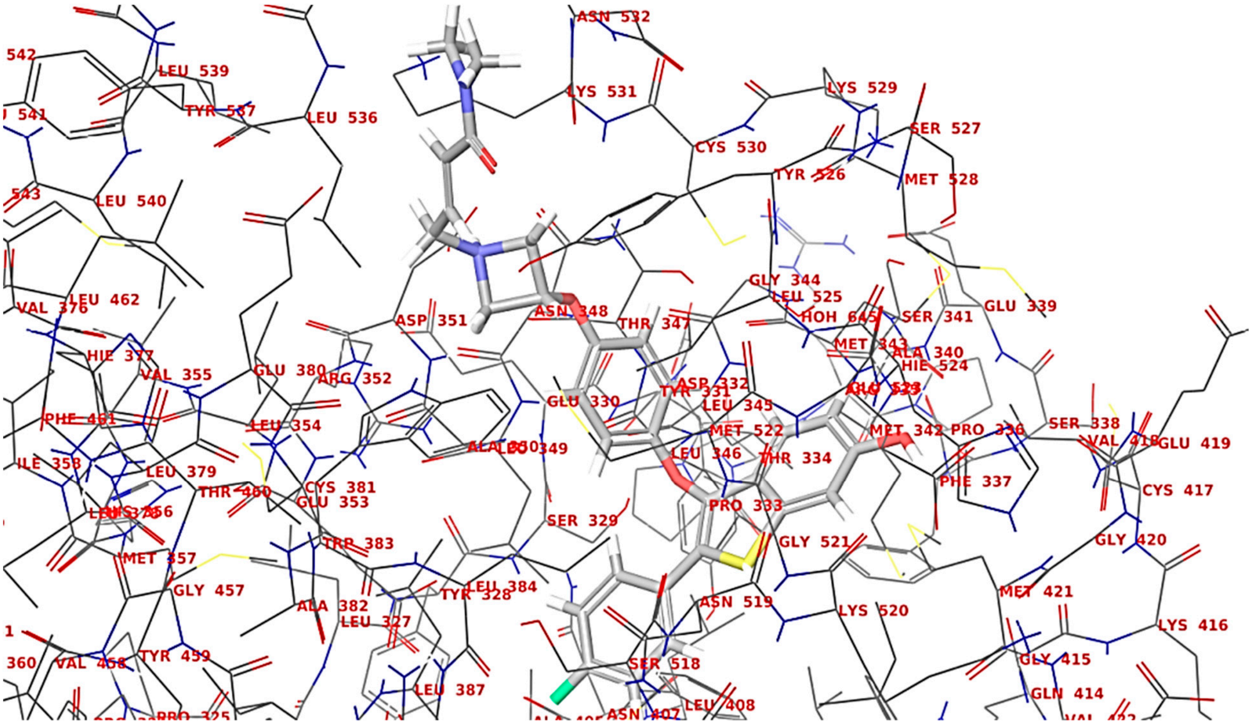

Figure S1b: 2D & 3D interactions for Compound BT_ER_21b (Docking score -12.577)

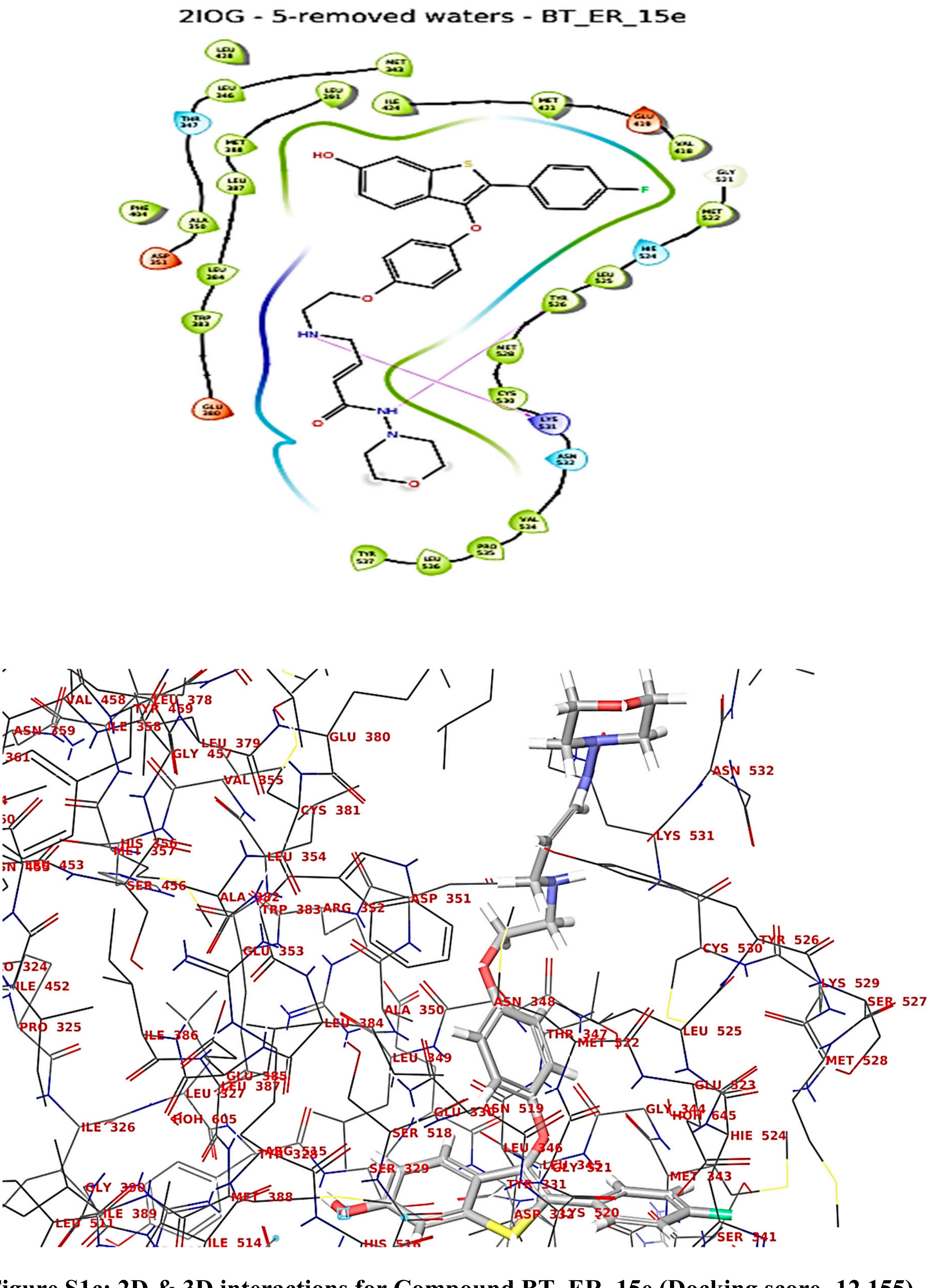

Figure S1c: 2D & 3D interactions for Compound BT_ER_15e (Docking score -12.155)

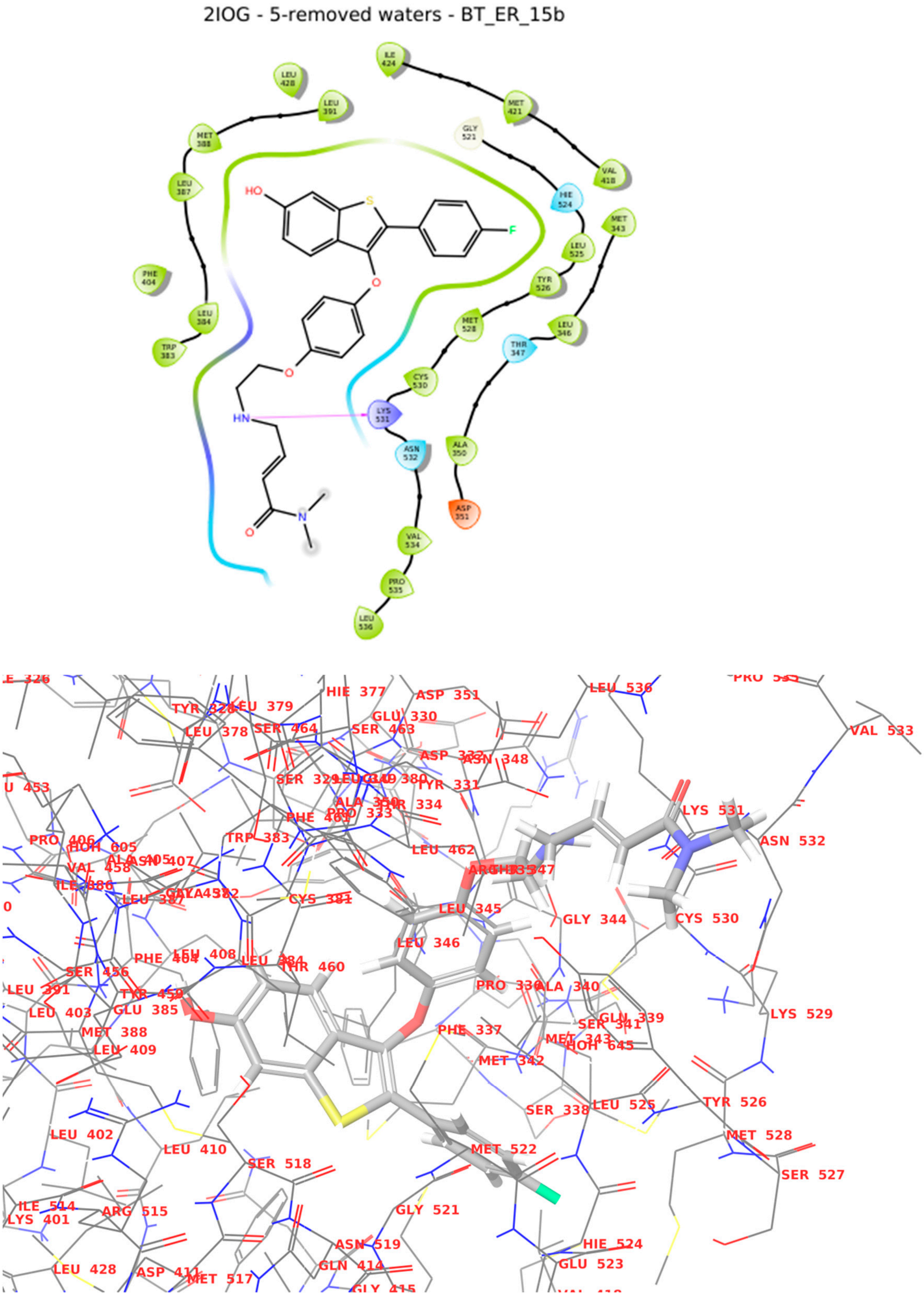

Figure S1d: 2D & 3D interactions for Compound BT_ER_15b (Docking score -12.007)

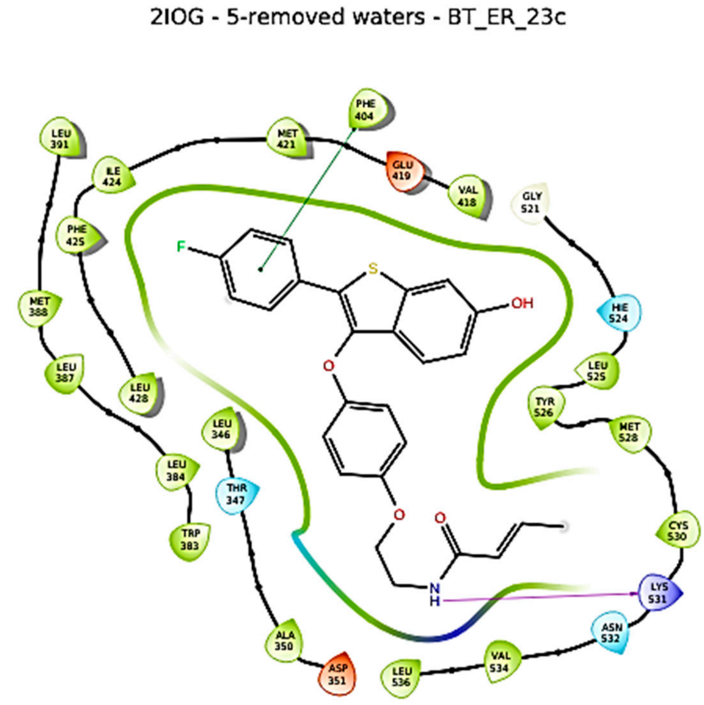

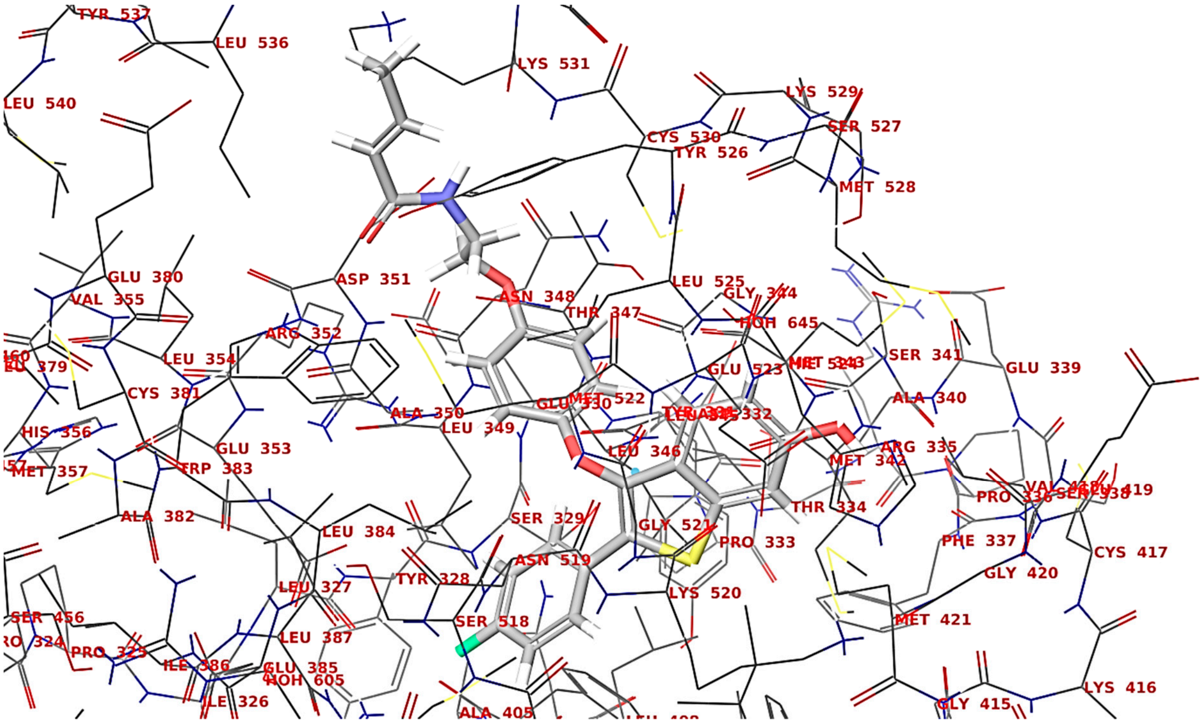

Figure S1e: 2D & 3D interactions for Compound BT_ER_23c (Docking score -12.394)

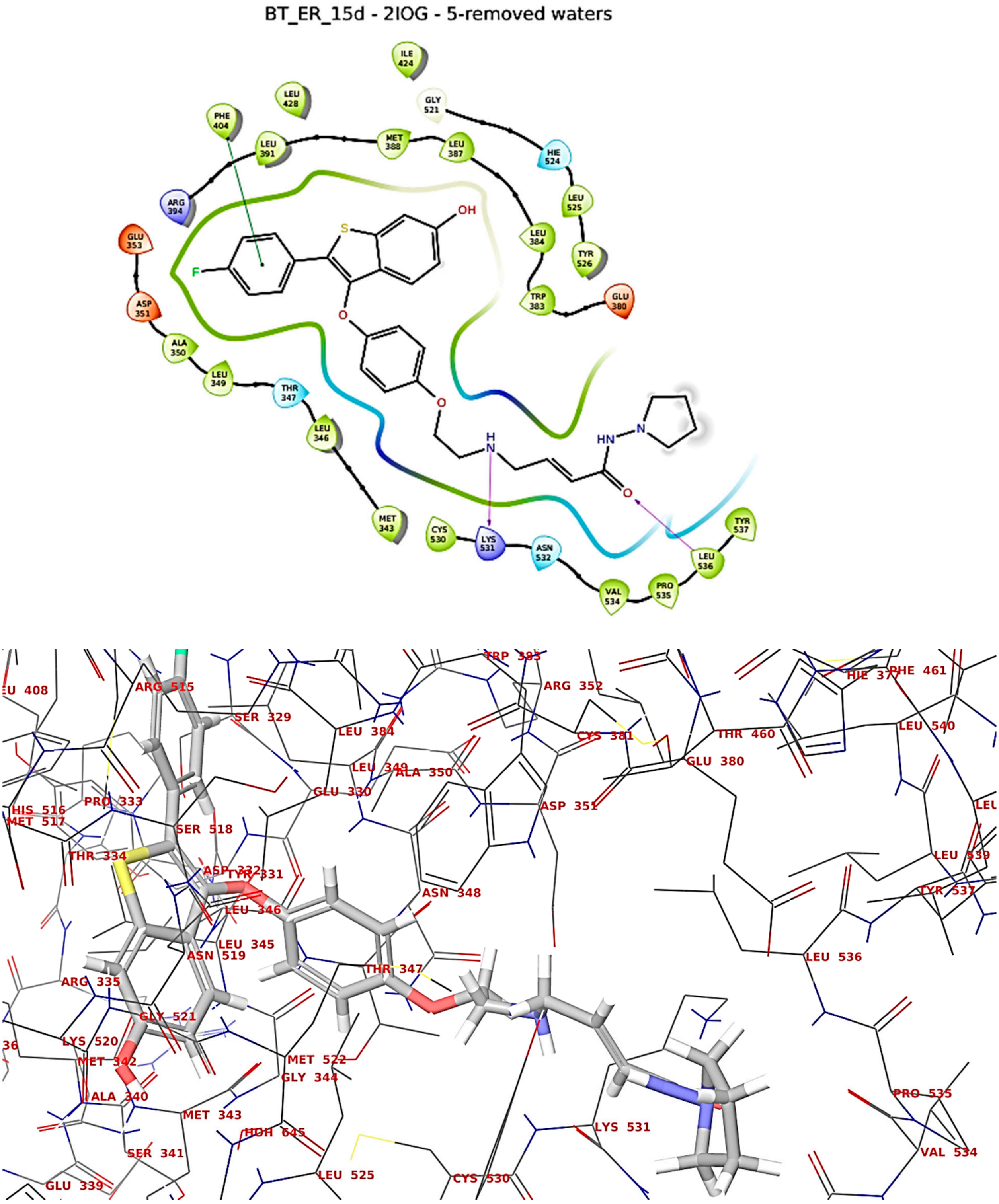

Figure S1f: 2D & 3D interactions for Compound BT_ER_15d (Docking score -11.524)

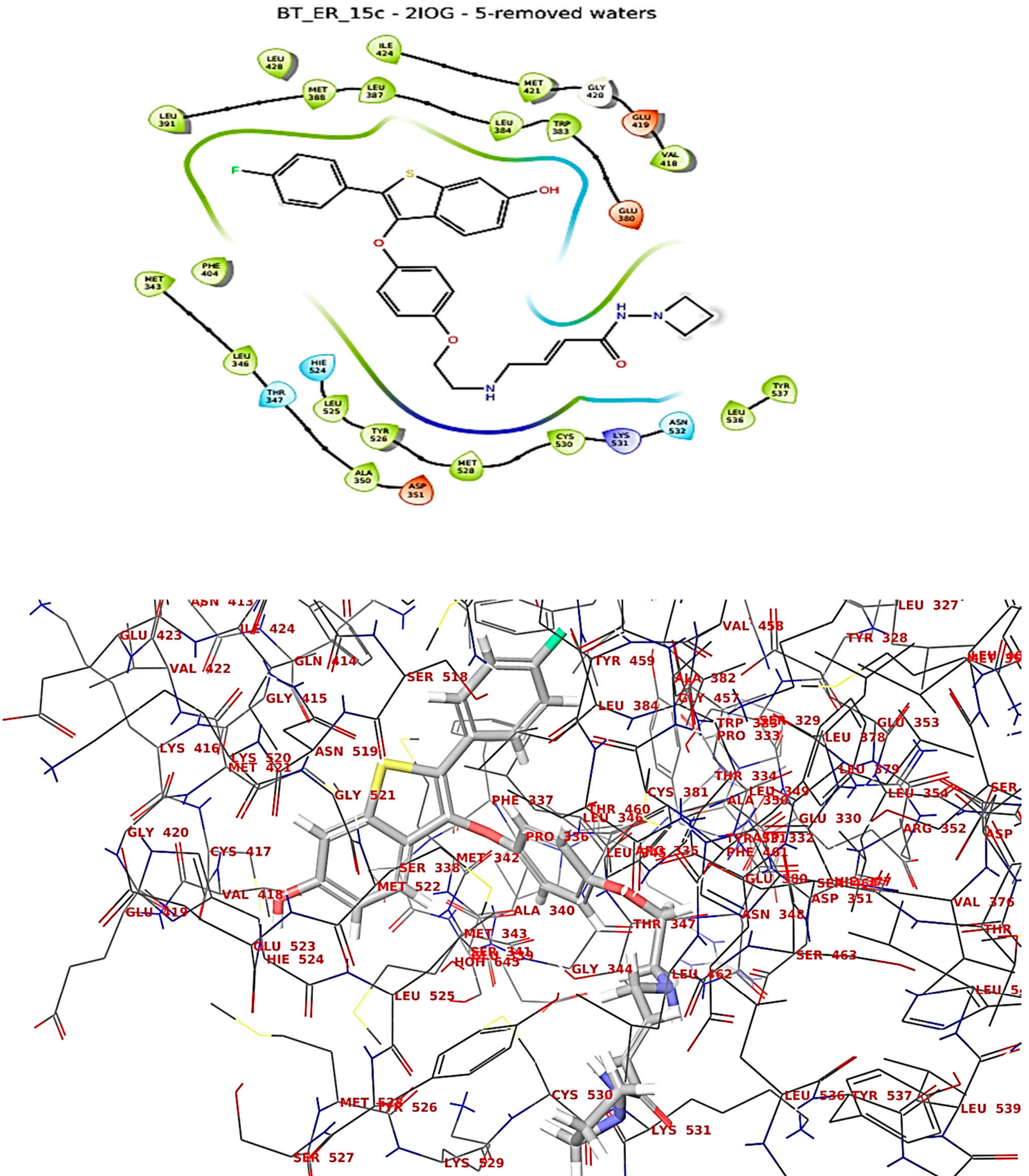

Figure S1g: 2D & 3D interactions for Compound BT_ER_15c (Docking score -11.459)

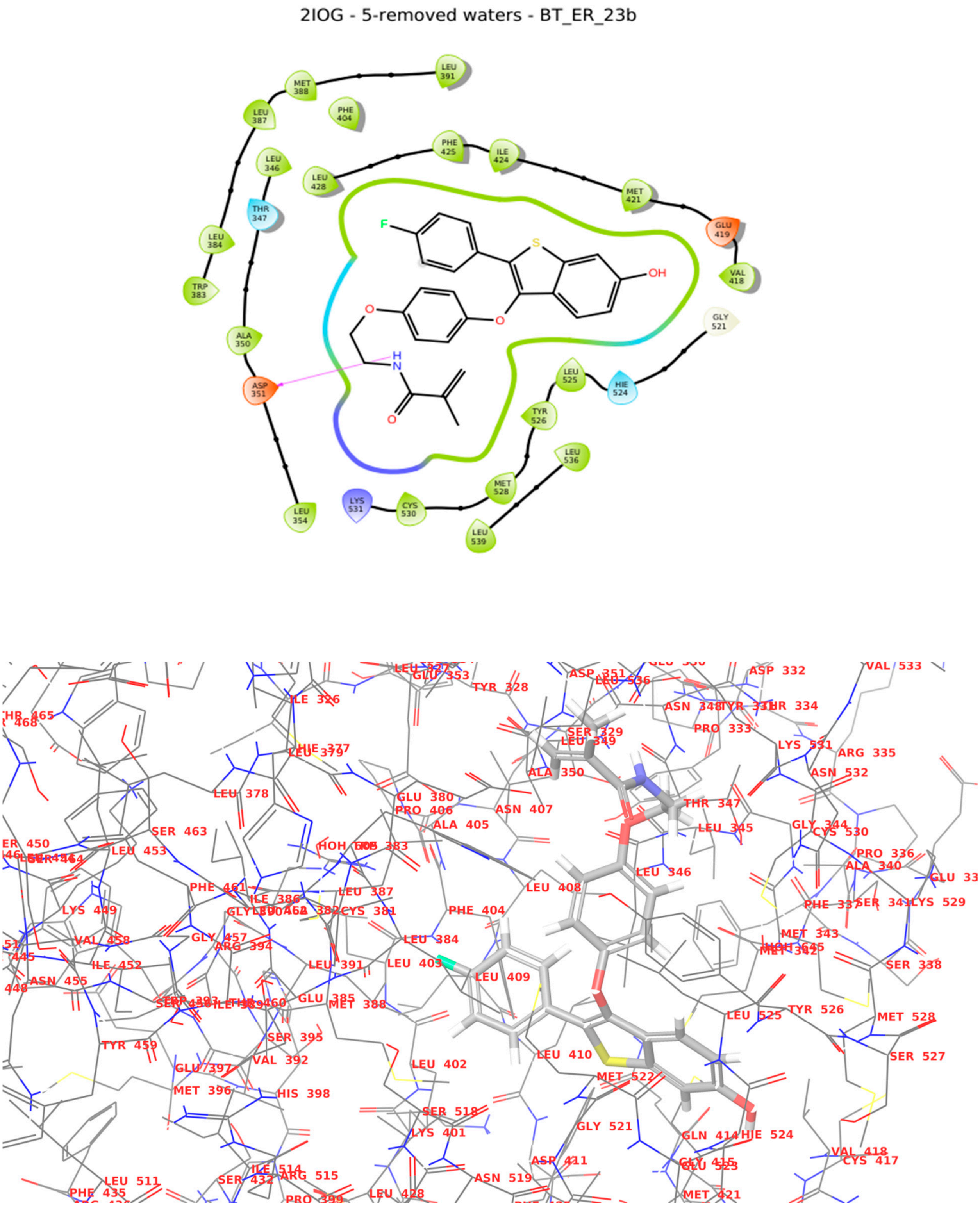

Figure S1h: 2D & 3D interactions for Compound BT_ER_23b (Docking score -11.622)

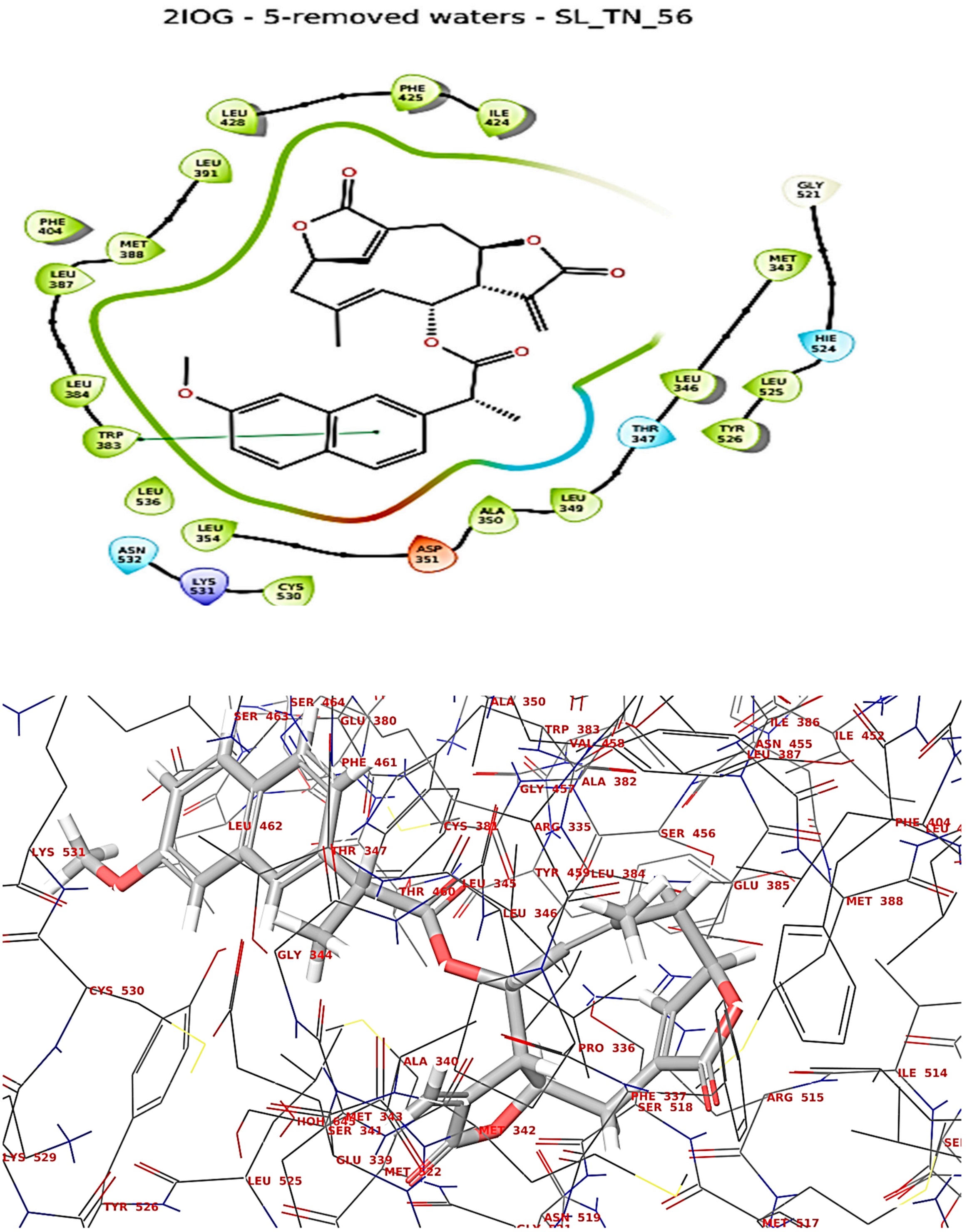

Figure S1i: 2D & 3D interactions for Compound SL_TN_56 (Docking score -10.962)

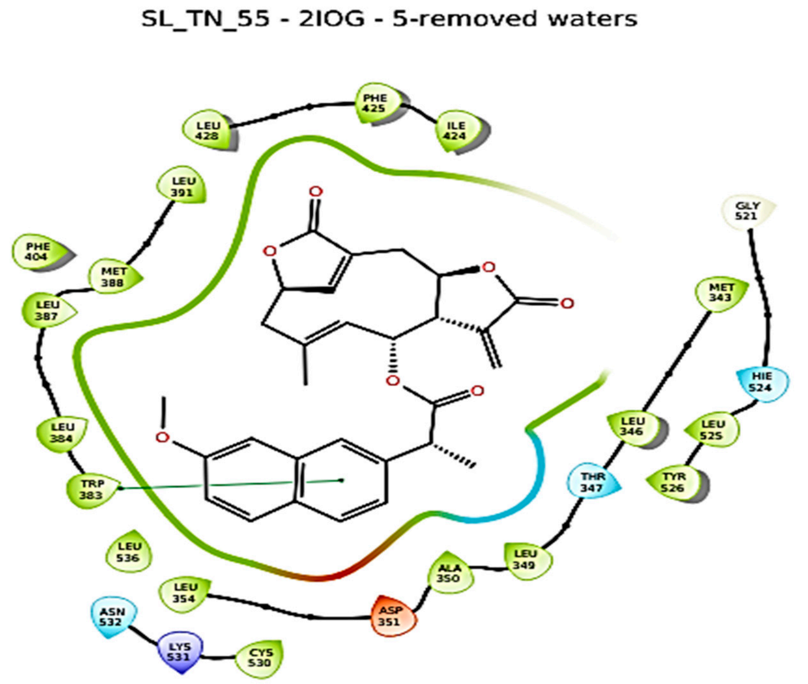

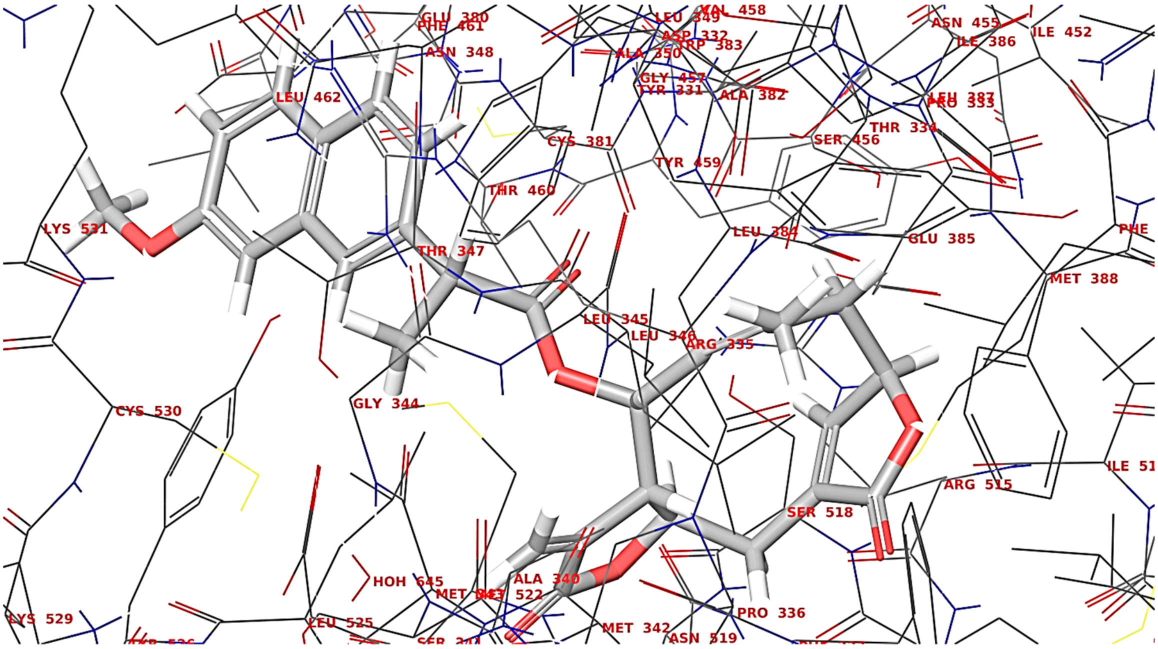

Figure S1j: 2D & 3D interactions for Compound SL_TN_55 (Docking score -10.962)
